# Hand-Based Gesture Recognition for Vehicular Applications Using IR-UWB Radar

**DOI:** 10.3390/s17040833

**Published:** 2017-04-11

**Authors:** Faheem Khan, Seong Kyu Leem, Sung Ho Cho

**Affiliations:** Department of Electronics and Computer Engineering, Hanyang University, 222 Wangsimini-ro, Seongdong-gu, Seoul 133-791, Korea; faheemkhan@hanyang.ac.kr (F.K.); cromy07@hanyang.ac.kr (S.K.L.)

**Keywords:** gesture recognition, IR-UWB radar, unsupervised learning, motion recognition, radar sensor, distance compensation, user interface

## Abstract

Modern cars continue to offer more and more functionalities due to which they need a growing number of commands. As the driver tries to monitor the road and the graphic user interface simultaneously, his/her overall efficiency is reduced. In order to reduce the visual attention necessary for monitoring, a gesture-based user interface is very important. In this paper, gesture recognition for a vehicle through impulse radio ultra-wideband (IR-UWB) radar is discussed. The gestures can be used to control different electronic devices inside a vehicle. The gestures are based on human hand and finger motion. We have implemented a real-time version using only one radar sensor. Studies on gesture recognition using IR-UWB radar have rarely been carried out, and some studies are merely simple methods using the magnitude of the reflected signal or those whose performance deteriorates largely due to changes in distance or direction. In this study, we propose a new hand-based gesture recognition algorithm that works robustly against changes in distance or direction while responding only to defined gestures by ignoring meaningless motions. We used three independent features, i.e., variance of the probability density function (pdf) of the magnitude histogram, time of arrival (TOA) variation and the frequency of the reflected signal, to classify the gestures. A data fitting method is included to differentiate between gesture signals and unintended hand or body motions. We have used the clustering technique for the classification of the gestures. Moreover, the distance information is used as an additional input parameter to the clustering algorithm, such that the recognition technique will not be vulnerable to distance change. The hand-based gesture recognition proposed in this paper would be a key technology of future automobile user interfaces.

## 1. Introduction

Hand-based gesture recognition is one of the hottest research fields, since it is of great significance in designing artificially intelligent human computer interfaces. Driving a modern car is an extremely difficult task [1]. A driver has to perform multi-tasking, such as observing the road, monitoring the vehicle’s status, Global Positioning System (GPS) monitoring, operating numerous electronic and mechanical devices and using audio entertainment. The gesture interface inside a car can assist the driver to perform various tasks. Different sensors have been used for gesture recognition, such as camera, radio-frequency identification (RFID), data-gloves, etc. [2,3,4,5,6,7,8,9]. Cameras, however, have a number of line of sight-related challenges that may prevent gesture recognition from being effective. For example, poorly-lit environments may have a negative impact on the image quality and in turn degrade the performance of gesture detection through the camera. The other main issue with camera-based gesture recognition is privacy [10]. An alternate method for gesture recognition is glove-based sensors. The data-glove-based methods use sensor devices for digitizing hand and finger motions into multi-parametric data [5]. The extra sensors make it easy to collect hand movement and configuration. However, the devices are quite expensive and bring much cumbersome experience to the users [6]. The environment inside a vehicle is usually dark at night, and it is inconvenient to wear something during driving; therefore, the above-mentioned techniques are not suitable for vehicular applications.

To overcome the above problems, radar-based gesture recognition can be used as a user interface inside a vehicle. Radar-based gesture recognition techniques have the advantage of better performance in dark environments, do not have privacy issues and do not require wearing sensors. In [11,12,13,14,15], the researchers have used Doppler radar sensors for gesture recognition. Molchanov et al. [11] used multiple sensors, including a depth camera and Doppler, for gesture recognition inside a vehicle. Portable radar sensors for gesture recognition in smart home applications are discussed in [12]. Kim Youngwook et al. [13] have performed hand-based gesture recognition using Doppler radar using machine learning techniques; however, the results are too dependent on the orientation and distance between hand and radar.

In addition to Doppler radars, UWB radars have been in the spotlight in recent years. There are many advantages of using IR-UWB radar, such as high range resolution and robustness to multipath due to the extremely wide bandwidth [16]. The major application areas of UWB radar technology are sensors and communications, localization, tracking and biomedical research [17]. IR-UWB sensor has been used in various radar applications, such as non-invasive vital sign monitoring [18,19,20], multiple object counting [21] and direction recognition of moving targets [22], and it has the ability to detect stationary or slowly moving targets [23]. However, there is very little reference work available in the literature about gesture recognition based on IR-UWB radar sensors. Ren Nan et al. [24] have presented an algorithm for big gesture recognition through IR-UWB radar, but the gestures detected in that work were simply based on the position difference of the hand and may not be useful in practical applications. Junbum Park et al. [25] used an IR-UWB radar sensor for detecting hand-based gestures through machine learning techniques. Although the results show high accuracy, there was an overfitting problem, and the gestures testing in a real environment showed much lower accuracy. Furthermore, there is no method included for the distance compensation or robustness of the algorithm to a change in distance or the orientation of the hand.

The main problem noted in the past radar-based gesture recognition algorithms was that they were vulnerable to distance and orientation; and the feature extraction through machine learning caused the overfitting problem in some cases, which made them error prone. To overcome these problems, we have presented a robust algorithm for hand-based gesture recognition using an IR-UWB radar sensor in this paper. We do not use the completely raw data as an input to the classifier in order to avoid the overfitting problem. We extracted three robust features, i.e., the variance of the pdf of the magnitude histogram, frequency and the variance of time of arrival (TOA) from the pre-processed signal reflected from the human hand. The features extracted were robust and showed better performance even if we changed the orientation of the hand. After the feature extraction, we used the K-means clustering algorithm for classification of the gestures. In order to make the algorithm robust against the distance and orientation variation, we have integrated the TOA-based distance information into the clustering algorithm.

**Figure 1:**
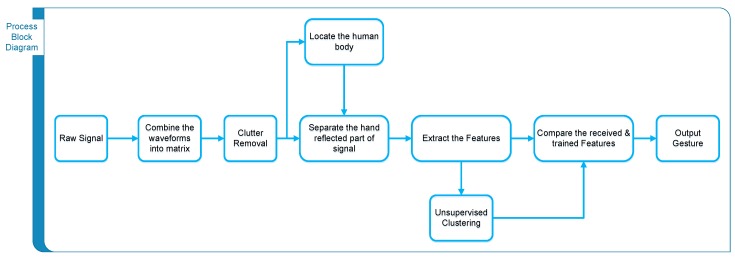
Process block diagram for gesture recognition.

In order to differentiate the gesture motion from some random hand or body motion, we included a data-fitting algorithm. Since the gesture motion defined in our work is almost periodic, therefore we fit the received gesture signal into a sinusoid and check the R-square value. If the R-square value is above a certain threshold, then it is supposed to be periodic and, hence, classified as a gesture signal; otherwise, it is classified as a non-gesture motion. The process block diagram of our algorithm is shown in Figure 1.

The main contribution of our work is that it is the first real-time IR-UWB-based gesture recognition technique, which avoids the overfitting problem and shows robustness when a change in distance or orientation of the hand occurs, because of the selection of robust parameters and the integration of the TOA information into the clustering algorithm. Additionally, we proposed an algorithm for the detection of only intended gestures while ignoring any random movement in front of the radar sensor. Considering these advantages, this method would be an important technology of the car user interface as one of the core technologies of the future autonomous vehicles.

**Figure 2:**
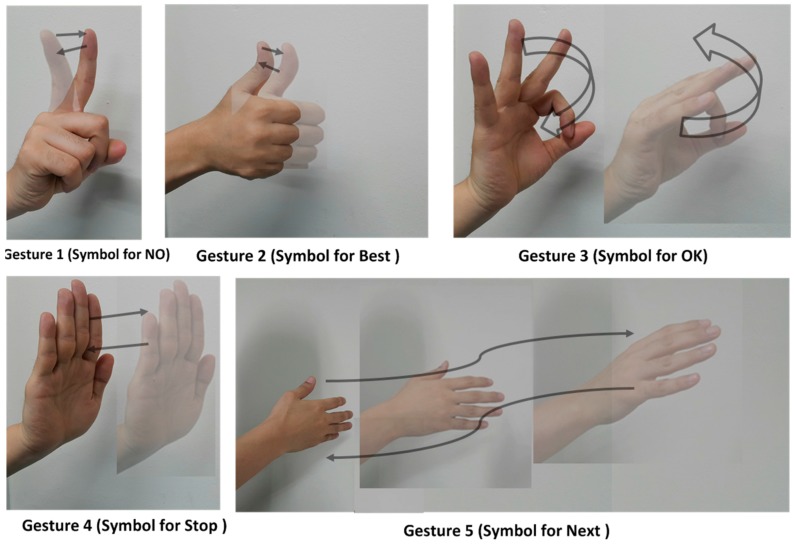
Gesture set for our experiments.

The hand-based gestures for our work are shown in Figure 2. The first gesture (Gesture 0) is the empty gesture when there is no hand movement in front of the radar. Table 1 shows the detailed explanation of the defined gestures. Gestures 1, 2 and 3 are broadly classified as small gestures, while Gestures 4 and 5 are classified as big gestures with larger displacements. The rest of the paper is organized as follows. In Section 2 of the paper, the feature extraction and classification are discussed. In Section 3, the results of gesture training and classification are presented, and conclusions are given in Section 4 of the paper. References are given at the end of the paper.

## 2. Feature Extraction and Classification

### 2.1. Signal Pre-Processing

**Figure 3:**
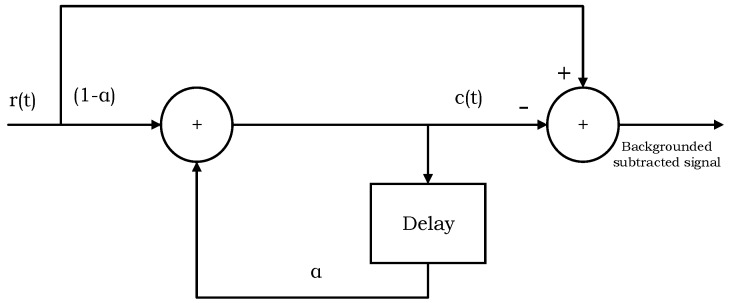
Clutter-removing filter (loopback filter).

From the raw signal reflected from the human hand, the clutter has to be removed. The loopback filter is used for removal of the clutter [26]. The loopback filter as represented by Figure 3 works as:

(1) $$c_{k}\left(t\right)=\propto c_{k-1}\left(t\right)+\left(1-\propto \right)r_{k}\left(t\right)$$

(2) $$y_{k}\left(t\right)=r_{k}\left(t\right)-c_{k}\left(t\right)$$

**Figure 4:**
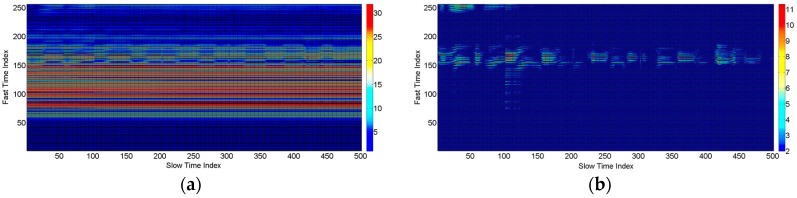
Gesture matrix (**a**) before clutter removal and (**b**) after clutter removal.

In the above equations, the symbol “∝” represents a constant used for weighting. For our experiments, the value of “∝” was 0.97. The symbol $c_{k}\left(t\right)$ represents the clutter signal, which is made until the *k*-th received sample. $y_{k}\left(t\right)$ is the background subtracted signal. From the above equations, it is clear that the new estimated clutter has two parts: one part is from the previous estimate, and one is from the current reflected signal. We need to store each filtered signal waveform and combine them into matrix $W_{mn}$ of size “m×n”. The “m” represents the slow time length, whereas the “n” represents the fast time length of the matrix. The “n” depends on the measurement distance or range of the radar. The slow time length “m” depends on the number of waveforms that we want to process at a single time. Since the gestures detected are all dynamic gestures, therefore the hand gesture area is detected and separated based on the maximum variance index of the signal in the fast time domain throughout the matrix duration “m”. The maximum variance index in the fast time shows the biggest change in the values over the gesture duration “m”, which we assume is the center of the location of the hand. We make the gesture matrix by combining the regions at the left and right side of the maximum variance index in the fast time domain. For example, in Figure 4, the gesture location is from Sample 140–Sample 190 in the fast time domain. The slow time length of the gesture matrix is determined by the gesture duration.

**Figure 5:**
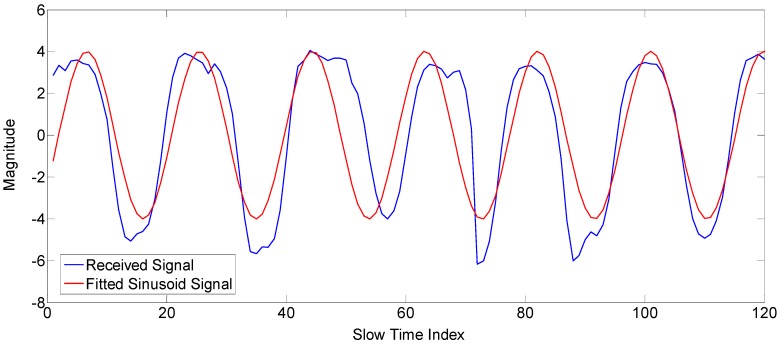
Gesture signal and the fitted sinusoid signal.

After we find the gesture matrix, we need to find whether the motion is due to the gesture signal or due to unintended hand motion. As the gestures defined in our work are periodic, so we use sinusoidal fitting to show how much the received data fit into the sinusoid. For the small gestures (Gestures 1, 2 and 3), the input data used for sinusoidal fitting are the magnitude data at the maximum variance index in the fast time index, as shown in Figure 5. However, for the big gestures (Gestures 4 and 5), the input data used for sinusoidal fitting are the TOA of each radar scan, as shown in Section 2.2.3. The R-square value is used for finding the fit of the signal, which is defined as follows.

(3) $$R^{2}=1-\frac{\sum_{i=1}^{n}{\left(y_{i}-{\hat{y}}_{i}\right)}^{2}}{\sum_{i=1}^{n}{\left(y_{i}-\bar{y}\right)}^{2}}$$

In Equation (3), “${\hat{y}}_{i}$” represent the estimated values of “$y_{i}$” by the fitting algorithm, whereas “$\bar{y}$” shows the mean of “$y_{i}$” [27]. The value of R-square lies between zero and one. The higher value of R-square shows that the prediction model is more accurate, and hence, the motion is due to the gesture signal, whereas the lower value of R-square shows unintended hand motion. The following Figure 5 shows the fitting algorithm result for the gesture signal. The resulting R-square value for the signal in the Figure 5 has some higher value, as it is a very accurate prediction model.

### 2.2. Features Extraction

The next step is to extract the features of interest from the gesture signal matrix. We extracted three features, i.e., the spread of the pdf of the gesture matrix histogram, the frequency of the hand gesture and the variance of the TOA of the gesture signal. The above three features are the parameters that can represent the characteristics of the human hand gesture. When an ordinary person carries out a repetitive hand gesture, each person has his or her own unique movements. This intrinsic motion is related to the movement range of the hand gesture, the speed of the hand gesture and the shape and size of the hand. The range of motion of the hand gesture is related to the variance of TOA; the speed of the hand gesture is related with frequency; and the shape and size of the hand are related to the spread of the magnitude histogram.

#### 2.2.1. Variance of the Magnitude Histogram

The magnitude histogram of the gesture matrix is found, and we use the data fitting technique to find the normal distribution pdf of the histogram. The variance “σ” of the resulting pdf is used as a feature for the classification of the gestures.

**Figure 6:**
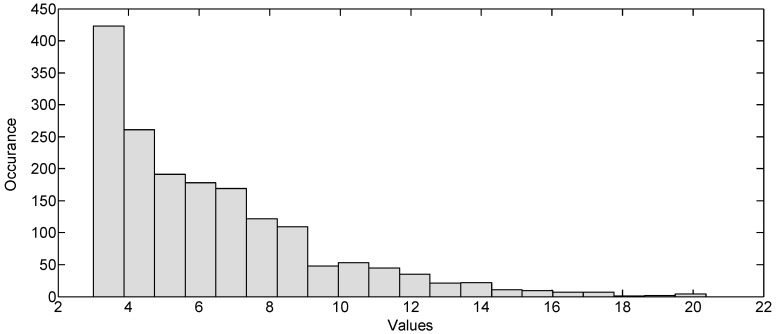
Magnitude histogram for a slow time of gesture duration.

**Figure 7:**
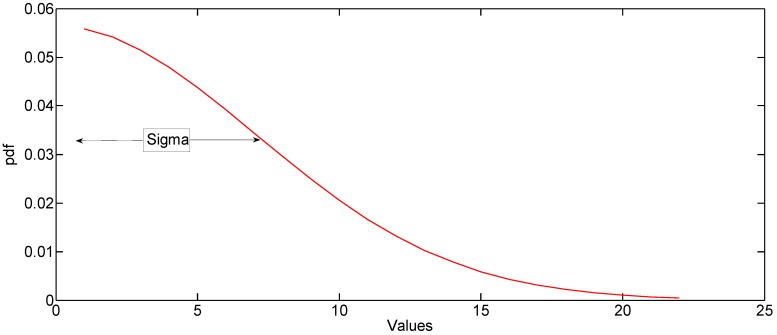
Pdf of magnitude histogram for a slow time of gesture duration.

In Figure 6, the magnitude histogram over the gesture duration is shown, and Figure 7 shows the pdf of the magnitude histogram. By using the pdf fitting method, the sigma value turns out to be a specific value. The sigma value is different for different gestures, as shown in the Results section. A large value of sigma means that the received signal has a higher magnitude over a certain period of time, which means a large hand gesture. Using the sigma method rather than simply using some received signal magnitudes makes the algorithm more robust because it statistically represents the magnitude characteristics of the reflected signal of each gesture over a certain period of time rather than the magnitude at a particular time or distance. The concrete method of calculating the spread of the histogram of the gesture matrix is shown in Algorithm 1.

**Algorithm 1.** Calculation of the spread of the histogram.Find the magnitude histogram of the gesture matrix, as shown in Figure 6.Although the small values appear the most in the histogram, we ignore those values, as these smaller values distort the shape of the histogram, and the most important values for classification of gestures are the higher values. We set the threshold on trial and error to discard the smaller values.Fit the resulting histogram to a normal pdf distribution, as shown in Figure 7. The histogram is considered the best way for density estimation, as discussed in reference [28].Find the spread “σ” of the normal pdf, as shown in Figure 7.

#### 2.2.2. Time of Arrival Variance

Time of arrival (TOA) variance represents the range of motion of a hand gesture. A hand gesture has a different moving distance for each hand gesture. A large hand gesture has a large TOA variance, and a small hand gesture has a small TOA variance. The gestures defined in our work have different TOA variances. The gesture set defined in our paper has five gestures, and two of the gestures have large variation in TOA, whereas the three gestures have very small variation of TOA. The specific method for the estimation of TOA is explained in Algorithm 2. The center of mass concept is used to find the centroid index of the waveform in Step 3 of Algorithm 2. The center of mass concept is a more robust feature because it reflects the characteristics of the entire waveform as compared to simply using the peak of the waveform as the centroid index.
**Algorithm 2.** TOA estimation.Find the index of the maximum variation column in slow time:
(4)$$n_{\max\_var}=argmax_{n\_fixed}\left|variation\left\{W_{mn}\left(:,n\_fixed\right)\right\}\right|$$
$W_{mn}$ is the gesture matrix, and the “m” represents the slow time length, whereas the “n” represents the fast time length of the matrix.Extract the $size_{fast}$ data around $n_{\max\_var}$ from one slow time scan data (the x axis is fast time, and the y axis is magnitude), and apply the Hilbert transform to obtain the envelope of these data.
(5)$$r_{hilbert}=abs\left[hilbert\left\{y\left(n_{\max\_var}-\frac{size_{fast}}{2}:n_{\max\_var}+\frac{size_{fast}}{2}\right)\right\}\right]$$
y represents the background-subtracted signal after the loopback filter in Section 2.1. $size_{fast}$ is basically determined by the length of Gaussian-modulated pulses transmitted and received and adds margins taking into account the slight length changes in Gaussian-modulated pulses that occur during reflection from the main target.Find the center of fast time index $n_{\mathrm{center}\_mass}$ using $r_{hilbert}$ and Equation (6).The equation below is similar to the center of mass concept:
(6)$$n_{\mathrm{center}\_mass}=\frac{\sum_{n=n_{\max\_var}-\frac{size_{fast}}{2}}^{n_{\max\_var}+\frac{size_{fast}}{2}}\;r_{hilbert}\left(n\right)*n}{\sum_{n=n_{\max\_var}-\frac{size_{fast}}{2}}^{n_{\max\_var}+\frac{size_{fast}}{2}}\;r_{hilbert}\left(n\right)}$$Find $n_{\mathrm{center}\_mass}$ using the method in Step 3 for each slow time.Find the TOA variance using $n_{\mathrm{center}\_mass}$ data in Step 4:
(7)$\mathrm{TOA}\;\mathrm{variance}=variance\left\{n_{\mathrm{center}\_mass}\left(1:m\right)\right\}$    The symbol “m” represents the slow time length.

#### 2.2.3. Frequency of the Gesture Signal

**Figure 8:**
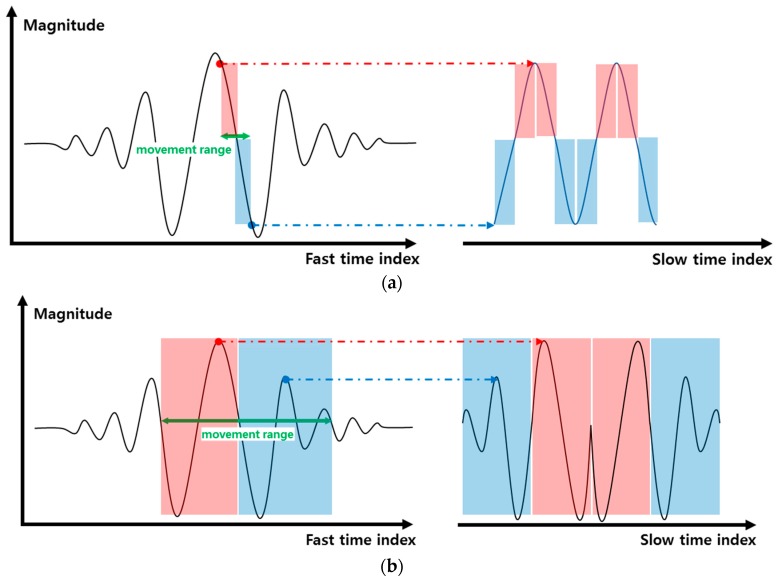

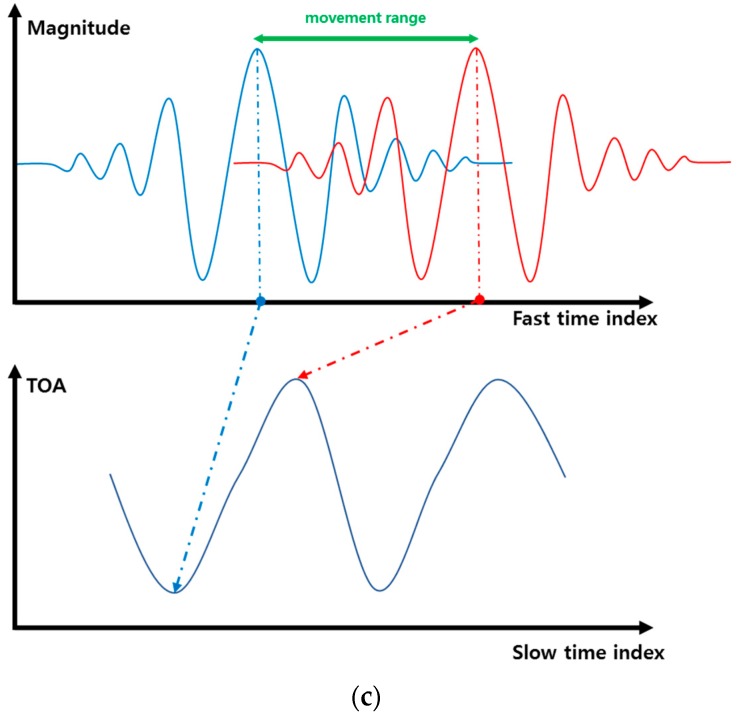
Illustrative explanation of the reason for introducing a new frequency extraction method based on TOA. (**a**) Slow time domain signal by the conventional magnitude-based method for a small gesture; (**b**) slow time domain signal by the conventional magnitude-based method for a big gesture; (**c**) slow time domain signal by the proposed TOA-based method for a big gesture.

Some hand gestures are fast, and the other hand gestures are relatively slow. This characteristic can be modeled through the frequency of the gesture signal. The other main purpose of introducing the frequency parameter is to distinguish between gestures and non-gesture motions of the body or some other change in the environment. We have defined two kinds of frequencies, i.e., frequency on the basis of magnitude variation and frequency based on the TOA variance. The magnitude-based spectrum is used for the small gestures, while the TOA frequency is used in the case of gestures that result in large displacement. The algorithm for obtaining frequency information using IR-UWB radar is widely used for the measurement of biological signals, such as respiration and pulse [18,19,20]. In these studies, frequency information was obtained by using the magnitude change of the slow time data at the fixed fast time point. In this study, frequency information was obtained by applying the same method to small gestures. However, in the case of a big gesture, the conventional method does not produce a satisfactory result. Figure 8 illustrates why existing methods do not work well in big gestures. First, as shown in Figure 8a, when the moving distance of the gesture is small, the waveform is similar to the sine wave. However, when the moving distance of the gesture is large, as shown in Figure 8b, a distorted waveform is generated reflecting the waveform of the modulated Gaussian pulse. Moreover, the real hand gestures do not have perfect periodicity, so the degree of distortion becomes worse. To solve this problem, we proposed a new frequency acquisition method based on TOA, as shown in Figure 8c. It estimates the optimum TOA for each slow time frame and predicts the frequency by observing the change of this TOA. The concrete method is given in Algorithm 3 as follows.

**Figure 9:**
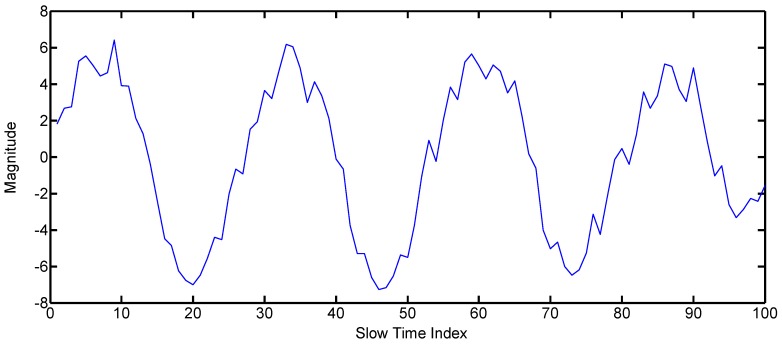
TOA of gesture with greater displacements (big gesture).

**Figure 10:**
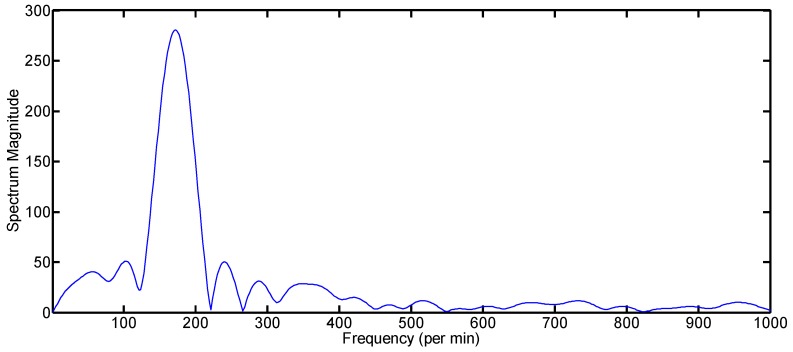
Spectrum of the TOA of gesture with greater displacements (big gesture).

**Algorithm 3.** Finding the frequency by the TOA-based method.Find the TOA of every column of the matrix *W_mn_* using the method given by Algorithm 2.Mean value subtraction: Find the mean of all of the TOA values and subtract it from each value. The resulting signal after mean subtraction is shown in Figure 9.Find the frequency domain signal by using the fast Fourier transform (FFT) algorithm.Search for the peak value of the spectrum as in Figure 10.The location of the peak value of the spectrum represents the frequency of the big gesture.

### 2.3. Gestures Classification

There are two broad classes of learning algorithms, i.e., supervised and unsupervised learning. In supervised learning, each output unit is told what its desired response to the input signals should be. However, in unsupervised learning, the algorithm is based on local information, and it does not know the target output for each input. It is also referred to as a self-organized network, as it self-organizes the data presented to the network and detects any collective properties in the data. In order to classify the gestures, the unsupervised learning algorithm (k-means clustering) is used. Clustering is a popular approach to implement the partitioning operation [29,30,31]. The clustering algorithm partitions a set of objects into clusters, such that the objects belonging to the same cluster have more similarity among themselves than with different clusters based on some defined criteria [32,33]. The main idea is to define K cluster centers. The next step is to associate each point of the given dataset to the nearest center. After that, we recalculate the centroids by taking the mean of the clusters. Now, a loop has been generated. We continue this process until the centers do not move their position any more. The cost function for the K-means algorithm is given by Equation (8).

(8) $$J\left(V\right)=\overset{c}{\underset{i=1}{\sum }}\;\;\overset{c_{i}}{\underset{j=1}{\sum }}{\left(∥x_{i}-v_{j}∥\right)}^{2}$$

where $∥x_{i}-v_{j}∥$ is the Euclidean distance between $x_{i}$ and $v_{j}$ and “$c_{i}$” is the number of data points in the i−th cluster, while “c” is number of cluster centers. The main advantage of the K-means algorithm is that it is fast, robust and very simple to understand. It gives the best result when the datasets are distinct and well separated.

**Figure 11:**
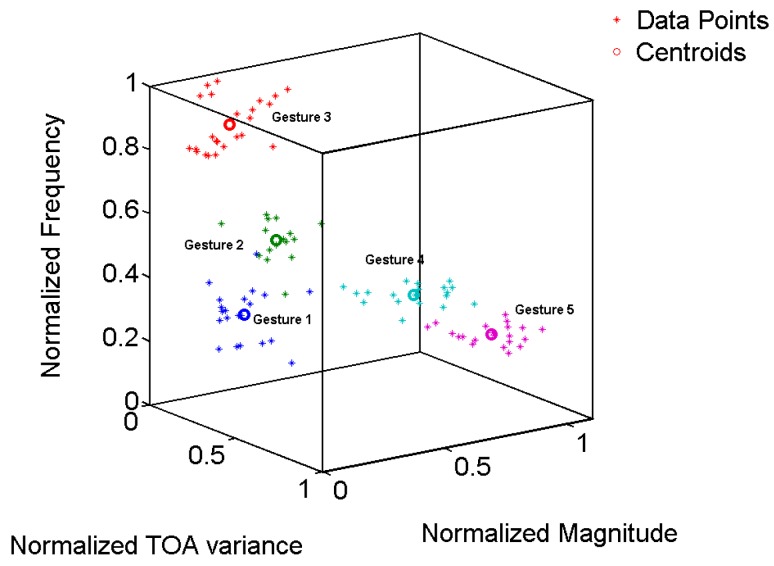
K-means clustering for gesture recognition.

In the case of our classification task, we train the algorithm by using the three input features as defined in Section 2.2 and then use the newly made gesture to find to which class it belongs. The number of centroids is the same as the number of gestures, i.e., five. The training result of the classification is shown in Figure 11, as follows.

**Figure 12:**
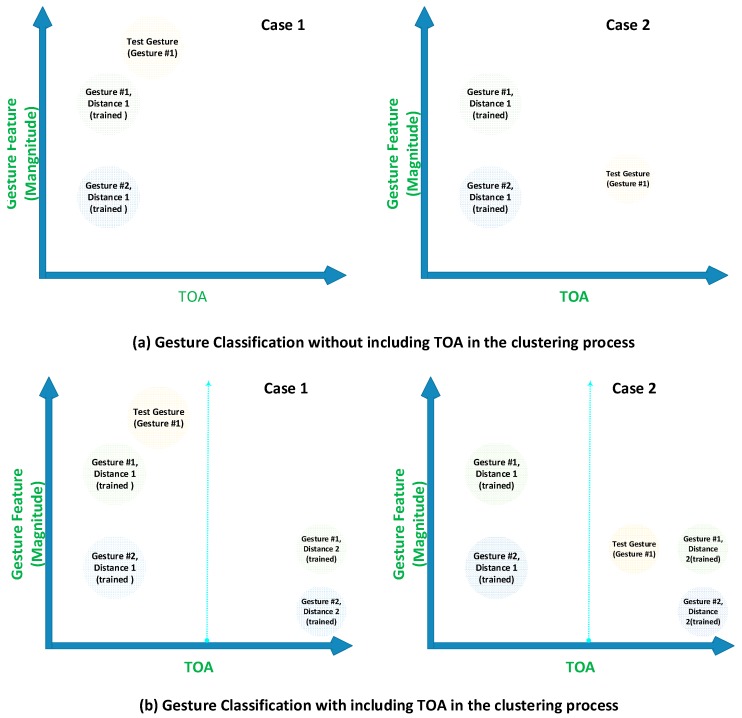
Logical explanation of clustering (**a**) without TOA information and (**b**) with TOA information.

In our work, we are focused on gesture recognition within some area (not just a fixed point) so that the driver can make gestures freely, which means that the training and testing locations for gestures might be different. Therefore, we need to compensate the distance change. To this end, we have proposed a clustering algorithm, which trains each gesture at two locations (nearest and farthest) and use the location information along with the three features defined in Section 2.2 as an input to the clustering algorithm. To explain the concept, we cannot show all four parameters on a plane surface; therefore, we used only magnitude and distance parameters for the explanation, as shown in Figure 12. In Figure 12, we used only two gestures for the explanation of our concept. The magnitude parameter changes inversely with distance. The clustering in Figure 12a has fixed point training, whereas Figure 12b has training at two points for every gesture, so it has two clusters for every gesture and uses the distance information to make decisions that are more robust.

As is clear from Figure 12a, if the TOA is not much different for the training and testing set, as in Case 1, then the gesture will be classified correctly as Gesture 1, as the test gesture has the nearest distance along the features’ axis to Gesture 1. However, if the training and testing set has much difference in TOA, as in Case 2, then the test gesture will be classified as Gesture 2 by the clustering algorithm because it has the nearest distance to Gesture 2 along the features’ axis. The reason for the incorrect decision in Case 2 is that training without TOA information with the algorithm does not take into account the decrease in magnitude of the signal with increasing TOA. In Figure 12b, every gesture is trained at two different distances, and for every gesture, two clusters are made at two different TOA locations. In Case 1 of Figure 12b, the test gesture is correctly classified, as it is nearest to the Gesture 1-trained cluster along the gesture features’ axis. In Case 2 of Figure 12b, the test gesture is located near the second set of clusters along the TOA line. The algorithm will check the TOA information along with the features’ information; therefore, it is classified as Gesture 1, because it is nearer to the second cluster of Gesture 1 as compared to the two clusters of Gesture 2. The gesture classification results of Figure 12 are shown clearly in Table 2 as follows.

## 3. Results and Discussion

### 3.1. Experimental Setup

**Figure 13:**
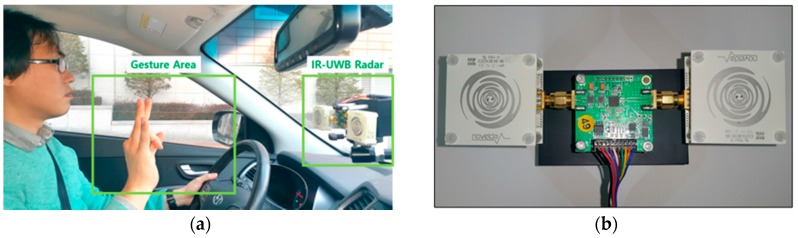
(**a**) Experimental setup inside the car; (**b**) IR-UWB radar.

The experimental setup for gesture recognition inside the car is shown in Figure 13a. The radar was placed in front of the driver, and the gestures were made by the right hand. The gesture area was almost 30 cm. Figure 13b shows the shape for the radar module. The transmit antenna and receiver antenna are connected to the IR-UWB transceiver, and a low noise amplifier (LNA) is mounted on the receiver to increase the reception performance.

In our experiments, we used the commercially available single-chip impulse radar transceiver (part number=NVA6201) made by NOVELDA (Novelda AS, Kviteseid, Norway). The parameter specifications are given in Table 3.

### 3.2. Feature Extraction Result

**Figure 14:**
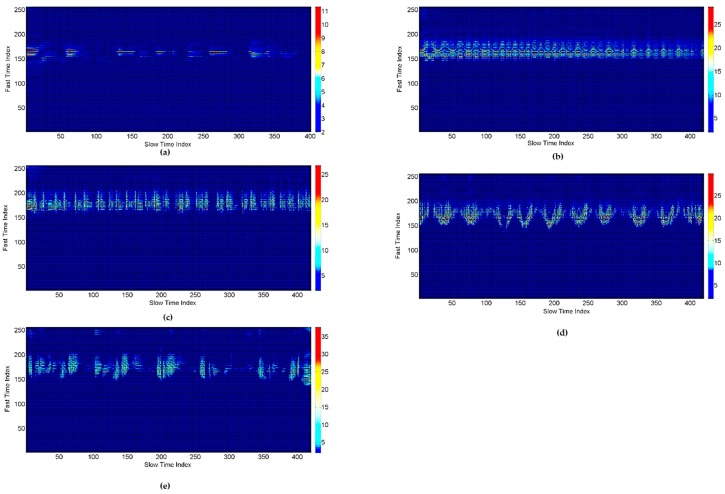
Gesture matrices after removing the clutter: (**a**) Gesture 1; (**b**) Gesture 2; (**c**) Gesture 3; (**d**) Gesture 4; (**e**) Gesture 5.

We removed the clutter from the gesture matrix. The gesture matrix for each gesture after removing the clutter is plotted in Figure 14.

**Figure 15:**
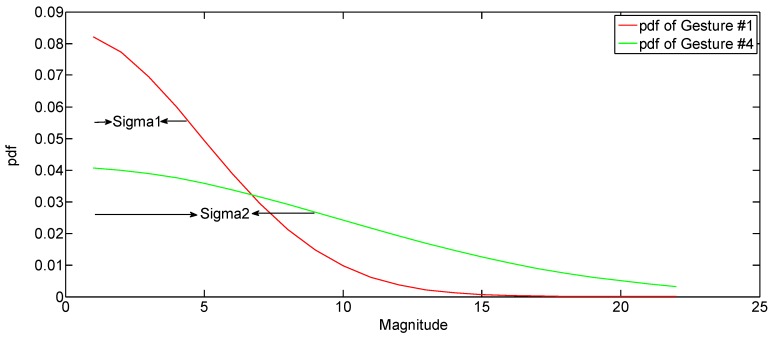
pdf graphs for small and big gestures.

After preprocessing the signal, the next step is to extract the features. The size of the matrix $W_{mn}$ for our experiments is (100 × 256), which means that the length of radar scans to be processed for each gesture is 100 (1.69 s), and the detection range is 256 samples or one meter. The pdf graphs for the small and big gestures are shown in Figure 15. It is clear from the figure that the small gesture has a low spread value as compared to the big gesture. The average spread values for all of the gestures defined in our work are shown in Table 4.

**Figure 16:**
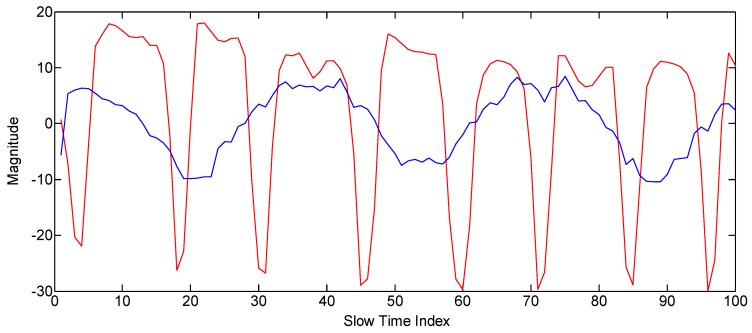
The red line represents the magnitude variation of Gesture 3, whereas the blue line represents the TOA variation of Gesture 4 across the slow time axis.

The sigma values for every pdf of the magnitude histogram are given in the second column of Table 4. The TOA variance is calculated by using the center of mass concept for finding the centroid index of the waveform. The third column of Table 4 shows the TOA variance result for all of the gestures averaged over the slow time. Another important feature is the frequency extraction. We conducted experiments to find the frequency for each gesture and found that the big gestures have relatively lower frequency as compared to the smaller gestures due to the greater displacements by the big gestures. The red line in Figure 16 shows the frequency information graph of Gesture 3 based on the magnitude of the signal, whereas the blue line in Figure 16 shows the frequency information graph of Gesture 4 based on the TOA variation. The frequency results for all gestures are given in Table 4.

From the results in Table 4, we noted that the big gestures (4 and 5) had relatively smaller frequency as compared to the smaller gestures. However, the frequency of each gesture depends on the user, and so, it can be determined during the training of gestures.

### 3.3. The Detection of Only Intended Gestures’ Result

The first important thing is to differentiate between the intended gesture motion and unintended random hand motion. To this end, we used the sinusoidal fitting algorithm, and the results of this algorithm are summarized in Table 5. We set the detection threshold of R-square as 0.2 for differentiating between gesture motion and undesired random motion.

Table 5 shows that the sinusoid data fitting technique resulted in 100 percentage accuracy for detecting the random motion, and hence, it can ignore the random hand motion, so that we can use only the useful gestures’ motion in the classification step. The number of trails for each gesture in Table 5 was 150, and we used three subjects (50 trails per subject).

### 3.4. Clustering Classification Results

From the features extraction section, it is clear that although some gestures overlap, the values for some particular parameters, however, did not overlap in all of the parameters. Therefore, we used the clustering technique in which we used all three parameters as inputs for the classification of the gestures. The K-means classifier is used to cluster the gestures. The input parameters are first normalized and then scaled before clustering by the K-means algorithm.

Figure 11 shows the sample result of the clustering in three dimensions by using the K-means algorithm. The K-means algorithm is very sensitive to the initialized values. In our experiments, we initialized the centroids for each gesture by using the mean values of each feature.

We conducted every gesture for 150 trials and three subjects (50 trials on one subject), calculated the percentage accuracy of every gesture and noted how much a gesture was overlapped by other gestures, which may result in the wrong detection. The results for the fixed point gesture training and gesture testing are shown in Table 6. The diagonal elements in the table represent that the original and classified gestures are the same. The off-diagonal elements represent the misdetected gestures.

As the driver’s hand may move in a certain area, which mean it can change its distance and orientation, therefore we used the training of the parameters at two distant points from the radar and integrated the distance information into the clustering algorithm.

As shown in Table 7, the performance of the algorithm deteriorates with the change in distance and orientation; therefore, we integrated the TOA information into the clustering algorithm. Table 8 below shows the results of the algorithm when distance compensation was used.

Finally, in Table 8, the results of training the gestures by using the three features and the TOA information are presented. By comparing the results of Table 8 with the results of Table 7, we can note that the diagonal elements in Table 8 have higher values as compared to Table 7, which means that our proposed clustering algorithm based on the three features, as well as TOA information is much more accurate than the algorithm without TOA information.

## 4. Conclusions

We have presented a robust algorithm for gesture recognition. We only used a single IR-UWB radar for our experiments. The three independent features showed better performance under different circumstances inside the vehicle. Although, if one parameter of a gesture overlapped with another gesture, sometimes, it was compensated by the other two parameters, resulting in an accurate result. The magnitude-based frequency was not very accurate for gestures with larger displacements; therefore, we defined another TOA-based frequency for the larger displacement gestures. We also integrated the TOA information along with the features’ information into the clustering algorithm, which resulted in much better performance, although the training and testing locations and orientations were not the same. The unintended motion created by randomly moving of the hands or body is nullified by using a data-fitting algorithm, and it showed accurate results. The confusion matrix showed that the results are very accurate within a certain area, which can cover the range of the driver’s hand motion, and therefore, the hand-based gesture recognition may be useful in practical applications to control electronic equipment inside any vehicle; hence, it can prove as a useful technology for the future user interface inside a vehicle.

## Figures and Tables

**Table 1:** Gestures defined for our work.

Gesture #	Explanation	Inference
**0**	When there is no hand movement in front of the radar	Empty Gesture
**1**	The finger is moving to the left and right slowly	NO
**2**	Thumbs up and the hand moving to and fro quickly, but with little displacements	BEST (Thumbs Up)
**3**	Three fingers going upward and downward while thumb and index fingers make an “O” symbol	OK
**4**	The hand palm is open and moving forward and backward with greater displacements while facing the radar transceiver	STOP
**5**	The hand palm is open and moving backward and forward diagonally with respect to the radar transceiver	NEXT

**Table 2:** Gesture classification results of Figure 12.

**Clustering without Taking TOA as Classification Input**		
**Case #**	Test Gesture (Original)	Classification Result
**01**	1	1
**02**	1	2
**Clustering with Taking TOA as Classification Input**		
**Case #**	Test Gesture (Original)	Classification Result
**01**	1	1
**02**	1	1

**Table 3:** Radar parameters’ values.

Parameter	Value
Centre Frequency	6.8 GHz
Pulse Repetition Frequency	100 MHz
Bandwidth	2.3 GHz
Output Power	−53 dBm/MHz
Slow Time Sampling Frequency	59 samples/s

**Table 4:** Magnitude histogram, TOA variance and Frequency results.

Gesture #	Spread of pdf of the Magnitude Histogram (Sigma)	TOA Variance	Frequency (per Minute)
**01**	4.2	1.9	57
**02**	5.7	3.1	115
**03**	7.3	2.1	96
**04**	11.3	5.5	62
**05**	9.1	4.9	49

**Table 5:** Data fitting results.

Gesture #	Mean (R-Square)	Detection Accuracy (% Age)
**Gesture #1**	0.62	100
**Gesture #2**	0.71	100
**Gesture #3**	0.61	100
**Gesture #4**	0.53	100
**Gesture #5**	0.40	100
**Hand moving for steering**	0.03	100
**Empty gesture**	0.01	100
**Body moving**	0.10	100
**Hand moving for gear change**	0.08	100
**Empty gesture**	0.02	100

**Table 6:** Fixed point training and testing results.

	Gesture #1	Gesture #2	Gesture #3	Gesture #4	Gesture #5
**Gesture #1**	100	0	0	0	0
**Gesture #2**	0	99.33	0.66	0	0
**Gesture #3**	0	0	100	0	0
**Gesture #4**	0	0	0.66	97.33	2
**Gesture #5**	0	0	0	2	98

**Table 7:** Training and testing at different distances (without distance compensation).

	Gesture #1	Gesture #2	Gesture #3	Gesture #4	Gesture #5
**Gesture #1**	90	5.33	4.66	0	0
**Gesture #2**	2.66	93.33	4	0	0
**Gesture #3**	2.66	4.66	92.66	0	0
**Gesture #4**	0	20.66	10.66	58.66	10.02
**Gesture #5**	8	18.66	6.66	20	46.66

**Table 8:** Training and testing at different distances and orientation (with distance compensation).

	Gesture #1	Gesture #2	Gesture #3	Gesture #4	Gesture #5
**Gesture #1**	98.66	0.66	0	0.66	0
**Gesture #2**	0	97.33	1.33	0	1.33
**Gesture #3**	0	0.66	99.33	0	0
**Gesture #4**	0	5.31	2.66	88.66	6
**Gesture #5**	0	4.01	0	4.66	91.33
